# Supplementary material for: The epidemiological risk factors of hand, foot, mouth disease among children in Singapore: A retrospective case-control study
Source: PLoS One. 2020 Aug 11;15(8):e0236711. doi: 10.1371/journal.pone.0236711 (PMC7418981; doi:10.1371/journal.pone.0236711)
Supplement: S1 File — (DOCX) [file pone.0236711.s001.docx]

**Subject ID:**

|  |  |  |  |  |
| --- | --- | --- | --- | --- |

Instructions:

1. Parents are preferred to complete the survey questionnaire.
2. Please tick ONE best response for each item below unless otherwise specified.

**Data Collection Form**

| **I. Child Demographics and Health Information** | |
| --- | --- |
| **Child gender** | ☐ Male  ☐ Female |
| **Date of birth** | \|  \|  \|  \|  \|  \|  \|  \|  \| \| --- \| --- \| --- \| --- \| --- \| --- \| --- \| --- \| \| D \| D \| M \| M \| Y \| Y \| Y \| Y \| |
| **Nationality** | ☐ Singaporean  ☐ Singapore permanent resident, please specify: _____________  ☐ Others, please specify: ________________________________ |
| **Ethnicity** | ☐ Chinese ☐ Malay ☐ Indian  ☐ Others, please specify: ________________________________ |
| **Child birth weight** | ☐ Less than 2.5kg ☐ 3.0 – 3.4kg ☐ 4.0 – 4.4kg  ☐ 2.5 – 2.9kg ☐ 3.5 – 3.9kg ☐ More than 4.4kg |
| **Mother’s gestational age** | ☐ Less than 37 weeks  ☐ 37 – 42 weeks |
| **Mode of delivery** | ☐ Natural birth ☐ Cesarean section  ☐ Others, please specify: ________________________________ |
| **Mother’s age at child birth** | ☐ Below 24 years old ☐ 35 – 39 years old  ☐ 25 – 29 years old ☐ Above 40 years old  ☐ 30 – 34 years old |
| **Breastfeeding history and pattern** | ☐ Never  ☐ Mixed breastfeed with infant formula  ☐ Exclusive breastfeed for 2 months  ☐ Exclusive breastfeed for 4 months  ☐ Exclusive breastfeed for 6 months or more |
| **Age of starting solids during infancy** | ☐ 4 – 6 months old  ☐ 7 – 9 months old  ☐ 10 – 12 months old  ☐ Above 13 months old |
| **History of HFMD/herpangina** | ☐ Never  ☐ Once, please specify: ☐ HFMD ☐ Herpangina  Year of infection: ___________________________________  ☐ More than once, please specify: ☐ HFMD ☐ Herpangina  Year of infection: ___________________________________  Frequency of infection: ­­­­­­­­­_______________________________ |
| **Hospitalisation due to HFMD/herpangina** | ☐ No  ☐ Yes, please specify year of hospitalisation: ________________ |
| **Number of siblings in the same childcare centre** | ☐ 0 ☐ 3  ☐ 1 ☐ 4  ☐ 2 ☐ 5 |
| **Siblings infected with HFMD/herpangina before** | ☐ No  ☐ Yes, please specify: ☐ HFMD ☐ Herpangina  Sibling’s gender: ☐ Male ☐ Female  Year of infection: ­­­­­­­­­___________________________________ |
| **Healthcare provider consulted**  *(may tick more than one)* | ☐ Private GP ☐ Polyclinic  ☐ Private paediatrician ☐ Public hospital  ☐ Private hospital ☐ Others, please specify: __________ |
| **Flu or influenza** *(fever, cold, cough, sore throat)*  **in the last 3 months** | ☐ No  ☐ Yes, please specify if hospitalized: ☐ No ☐ Yes |
| **Diarrheal** *(3 or more times water stool over 24hr)*  **in the last 3 months** | ☐ No  ☐ Yes, please specify if hospitalized: ☐ No ☐ Yes |
| **History of other diseases** | ☐ None  ☐ Chicken pox  ☐ Mumps  ☐ Others, please specify: ________________________________ |
| **Other health concerns** | ☐ None  ☐ Asthma  ☐ G6PD deficiency  ☐ Epilepsy  ☐ Eczema  ☐ Food allergy, please specify food: _______________________  ☐ Drug allergy, please specify drug: _______________________  ☐ Others, please specify: ________________________________ |
| **Long term medication** | ☐ No  ☐ Yes, please specify medical condition: ___________________ |
| **Optional vaccination taken before**  *(may tick more than one)* | ☐ None  ☐ Influenza/Flu ☐ Pneumococcal  ☐ Chicken pox ☐ Haemophilus Influenza type B (Hib)  ☐ Rotavirus ☐ Others, please specify: ____________ |
| **Main caretaker when child is sick** | ☐ Parents  ☐ Grandparents  ☐ Helper/maid  ☐ Neighbour  ☐ Close relatives, please specify: _________________________  ☐ Others, please specify: ________________________________ |

| **II. Parent Demographics and Health Information** | |
| --- | --- |
| **Relationship to child** | ☐ Mother ☐ Father  ☐ Others, please specify: ________________________________ |
| **Mother’s age** | ☐ 21–30 years old ☐ 41–50 years old ☐Above 61 years old  ☐ 31–40 years old ☐ 51–60 years old |
| **Father’s age** | ☐ 21–30 years old ☐ 41–50 years old ☐Above 61 years old  ☐ 31–40 years old ☐ 51–60 years old |
| **Total number of children** | ☐ 1 ☐ 3 ☐ 5  ☐ 2 ☐ 4 ☐ Others, please specify: ___ |
| **Household size** | ☐ 2 ☐ 4 ☐ 6  ☐ 3 ☐ 5 ☐ Others, please specify: ___ |
| **Household family members** | ☐ Parents and child  ☐ Parents, child and helper/maid  ☐ Grandparents, parents and child  ☐ Grandparents, parents, child and helper/maid  ☐ Others, please specify: ________________________________ |
| **Highest education level** | ☐ No formal education  ☐ Primary  ☐ Secondary  ☐ Tertiary  ☐ Others, please specify: ________________________________  Number of years of education received: ____________________ |
| **Housing type** | ☐ Public housing: HDB  ☐ Private housing: Condominiums, apartments, landed properties  ☐ Others, please specify: ________________________________ |
| **Housing space** | ☐ 1 to 3 rooms ☐ 4 to 5 rooms  ☐ More than 5 rooms, please specify: ______________________ |
| **Average gross household monthly income from work** | ☐ Below S$2,000 ☐ S$6,000 – $8,000  ☐ S$2,000 – $4,000 ☐ S$8,000 – $10,000  ☐ S$4,000 – $6,000 ☐ Above S$10,000 |
| **Any of the parents has diabetes?** | ☐ None ☐ Both  ☐ Mother ☐ Father |
| **Any of the parents has hypertension?** | ☐ None ☐ Both  ☐ Mother ☐ Father |
| **Any of the parents has hypercholesterolemia?** | ☐ None ☐ Both  ☐ Mother ☐ Father |
| **Any of the parents has HFMD/herpangina before?** | ☐ None ☐ Both  ☐ Mother ☐ Father |
| **Average number of days of work leave taken when child had HFMD/herpangina** | ☐ 1 day ☐ More than 5 days  ☐ 2 – 4 days ☐ 0 day Reason:________________ |
| **Average money spent per medical consultation when child had HFMD/herpangina** | ☐ Below S$50 ☐ Above S$100  ☐ S$51 – S$100 ☐ S$0 Reason:________________ |

| **III. Risk Factors** | | |
| --- | --- | --- |
| **Child plays in outdoor playground** | ☐ Never  ☐ Once every 2 to 3 weeks  ☐ Once a week  ☐ More than once a week  ☐ Everyday | |
| **Child plays in indoor playground** | ☐ Never  ☐ Once every 2 to 3 weeks  ☐ Once a week  ☐ More than once a week  ☐ Everyday | |
| **Child plays with other children in the neighbourhood** | ☐ Never  ☐ Once every 2 to 3 weeks  ☐ Once a week  ☐ More than once a week  ☐ Everyday | |
| **Child washes hands with soap before eating** | ☐ Never  ☐ Sometimes  ☐ Always | |
| **Child washes hands with soap after eating** | ☐ Never  ☐ Sometimes  ☐ Always | |
| **Child washes hands with soap after toilet** | ☐ Never  ☐ Sometimes  ☐ Always | |
| **Adult washes hands with soap after changing diaper or washing up child after toilet** | ☐ Never  ☐ Sometimes  ☐ Always | |
| **Adult washes hands with soap before feeding child** | ☐ Never  ☐ Sometimes  ☐ Always | |
| **Use hand sanitiser for child when dining outside** | ☐ Never  ☐ Sometimes  ☐ Always | |
| **Use sanitiser for baby seats when dining outside** | ☐ Never  ☐ Sometimes  ☐ Always | |
| **Wash toys with soap at home** | ☐ Never  ☐ Once every 2 to 3 weeks  ☐ Once a week  ☐ More than once a week  ☐ Everyday | |
| **Use sanitiser to clean toys at home** | ☐ Never  ☐ Once every 2 to 3 weeks  ☐ Once a week  ☐ More than once a week  ☐ Everyday | |
| **Household cleaning frequency** | ☐ Never  ☐ Once every 2 to 3 weeks  ☐ Once a week  ☐ More than once a week  ☐ Everyday | |
| **Share utensils with siblings at home** | ☐ Never  ☐ Sometimes  ☐ Always | |
| **IV. Knowledge of HFMD** | | |
| **HFMD is transmitted from an infected person to a healthy individual through direct contact with nasal secretions (such as saliva, sputum or mucus), fluids from blister or rash, and feces.** | | ☐ No  ☐ Yes  ☐ Don’t know |
| **Fever, mouth ulcers, rash or blisters on palms, soles, and/or buttocks are some common signs and symptoms of HFMD.** | | ☐ No  ☐ Yes  ☐ Don’t know |
| **Incubation period (period from infection to onset of symptoms) of HFMD is usually 3 to 5 days and range from 2 days to 2 weeks.** | | ☐ No  ☐ Yes  ☐ Don’t know |
| **Adults can get infected with HFMD.** | | ☐ No  ☐ Yes  ☐ Don’t know |
| **Children below age of five are at higher risk of HFMD infection.** | | ☐ No  ☐ Yes  ☐ Don’t know |
| **Keep child at home and stay away from public places when child is infected with HFMD.** | | ☐ No  ☐ Yes  ☐ Don’t know |
| **Good personal hygiene is important to prevent HFMD.** | | ☐ No  ☐ Yes  ☐ Don’t know |
| **There is no specific treatment for HFMD besides relief of symptoms.** | | ☐ No  ☐ Yes  ☐ Don’t know |
| **There is no HFMD vaccine currently available.** | | ☐ No  ☐ Yes  ☐ Don’t know |
| **Inform your child’s childcare centre immediately when child is diagnosed with HFMD.** | | ☐ No  ☐ Yes  ☐ Don’t know |
| **Child returns back to childcare centre only after the medical certificate issued by the doctor has expired and child has no more symptoms of HFMD.** | | ☐ No  ☐ Yes  ☐ Don’t know |
| **Not sharing food/drinks, eating utensils, toothbrushes or towels with others to prevent transmission of HFMD.** | | ☐ No  ☐ Yes  ☐ Don’t know |
| **Proper disinfection of toys or appliances contaminated by nasal or oral secretions.** | | ☐ No  ☐ Yes  ☐ Don’t know |
| **Shedding of HFMD can last for up to 12 weeks in stool even when the child has recovered.** | | ☐ No  ☐ Yes  ☐ Don’t know |
| **Shedding of HFMD can last for up to 4 weeks in saliva even when the child has recovered.** | | ☐ No  ☐ Yes  ☐ Don’t know |

**---------------------------------------------------------END-----------------------------------------------------------**

**编号:**

|  |  |  |  |  |
| --- | --- | --- | --- | --- |

指示:

1. 由家长填写此调查问卷为佳。
2. 请为以下每个项目选择一个最佳回复，除非另有指示。

**数据收集表**

| **I. 孩子背景与健康资料** | |
| --- | --- |
| **孩子性别** | ☐ 男  ☐ 女 |
| **出生日期** | \|  \|  \|  \|  \|  \|  \|  \|  \| \| --- \| --- \| --- \| --- \| --- \| --- \| --- \| --- \| \| 日 \| 日 \| 月 \| 月 \| 年 \| 年 \| 年 \| 年 \| |
| **国籍** | ☐ 新加坡公民  ☐ 新加坡永久居民, 请注明: ____________________________________  ☐ 其他, 请注明: ______________________________________________ |
| **种族** | ☐ 华人 ☐ 马来人 ☐ 印度人  ☐ 其他, 请注明: ______________________________________________ |
| **出生体重** | ☐ 2.5公斤以下 ☐ 3.0 – 3.4公斤 ☐ 4.0 – 4.4公斤  ☐ 2.5 – 2.9公斤 ☐ 3.5 – 3.9公斤 ☐ 4.4公斤以上 |
| **母亲的孕龄** | ☐ 少于37周  ☐ 37 – 42 周 |
| **分娩方式** | ☐ 自然分娩 ☐ 剖腹产  ☐ 其他, 请注明: ______________________________________________ |
| **母亲分娩时的年龄** | ☐ 24岁以下 ☐ 35 – 39岁  ☐ 25 – 29 岁 ☐ 40岁以上  ☐ 30 – 34岁 |
| **哺乳历史与模式** | ☐ 无  ☐ 混合母乳喂养和婴儿配方奶粉  ☐ 纯母乳喂养2个月  ☐ 纯母乳喂养4个月  ☐ 纯母乳喂养6个月或以上 |
| **婴儿时期开始吃固体食物的年龄** | ☐ 4 – 6个月大  ☐ 7 – 9个月大  ☐ 10 – 12个月大  ☐ 13个月大以上 |
| **手足口症/疱疹性咽峡炎的 病史** | ☐ 无  ☐ 一次, 请注明: ☐ 手足口症 ☐ 疱疹性咽峡炎 受感染的年份: _______  ☐ 多于一次, 请注明: ☐ 手足口症 ☐ 疱疹性咽峡炎  受感染的年份: _____________________________________________  受感染的次数: ­­­­­­­­­_____________________________________________ |
| **手足口症/疱疹性咽峡炎住院治疗** | ☐ 无  ☐ 有, 请注明年份: ____________________________________________ |
| **同一所幼儿中心的兄弟姐妹人数** | ☐ 0 ☐ 2 ☐ 4  ☐ 1 ☐ 3 ☐ 5 |
| **兄弟姐妹曾感染手足口症/疱疹性咽峡炎** | ☐ 无  ☐ 有, 请注明: ☐ 手足口症 ☐ 疱疹性咽峡炎  兄弟姐妹的性别: ☐ 男 ☐ 女  受感染的年份: _____________________________________________ |
| **医疗咨询提供者**  *(****可勾选多个****)* | ☐ 私人全科医生 ☐ 私人医院 ☐ 公立医院  ☐ 私人儿科医生 ☐ 综合诊所 ☐ 其他, 请注明: ____________ |
| **过去3个月内感染流感（发烧，感冒，咳嗽，喉咙痛）** | ☐ 无  ☐ 有, 请注明有无住院治疗:_____________________________________ |
| **过去3个月内有腹泻（24小时内3次或以上液体粪便）** | ☐ 无  ☐ 有, 请注明有无住院治疗:_____________________________________ |
| **其他病史** | ☐ 无  ☐ 水痘  ☐ 腮腺炎/流腮（猪头皮）  ☐ 其他, 请注明: ______________________________________________ |
| **其他健康问题** | ☐ 无  ☐ 哮喘  ☐ 葡萄糖六磷酸去氫酵素缺乏症/蠶豆症(G6PD缺乏症)  ☐ 癫痫  ☐ 湿疹  ☐ 食物敏感, 请注明食物: ______________________________________  ☐ 药物敏感, 请注明药物: ______________________________________  ☐ 其他, 请注明: ______________________________________________ |
| **长期服药** | ☐ 无  ☐ 有, 请注明病症: ____________________________________________ |
| **选择性疫苗注射历史**  *(****可勾选多个****)* | ☐ 无  ☐ 流感 ☐ 肺炎球菌  ☐ 水痘 ☐ B型流感嗜血杆菌 (Hib)  ☐ 轮状病毒 ☐ 其他, 请注明: _______________________________ |
| **孩子生病时的主要看护人** | ☐ 父母  ☐ 祖父母  ☐ 帮佣  ☐ 邻居  ☐ 近亲, 请注明: ______________________________________________  ☐ 其他, 请注明: ______________________________________________ |

| **II. 父母背景与健康资料** | |
| --- | --- |
| **与孩子的关系** | ☐ 母亲 ☐ 父亲  ☐ 其他, 请注明: ______________________________________________ |
| **母亲的年龄** | ☐ 21–30 岁 ☐ 51–60岁  ☐ 31–40岁 ☐ 61岁以上  ☐ 41–50岁 |
| **父亲的年龄** | ☐ 21–30 岁 ☐ 51–60岁  ☐ 31–40岁 ☐ 61岁以上  ☐ 41–50岁 |
| **孩子人数** | ☐ 1 ☐ 3 ☐ 5  ☐ 2 ☐ 4 ☐ 其他, 请注明: _________________ |
| **家庭人数** | ☐ 2 ☐ 4 ☐ 6  ☐ 3 ☐ 5 ☐ 其他, 请注明: _________________ |
| **家庭成员** | ☐ 父母与孩子  ☐ 父母, 孩子与帮佣  ☐ 祖父母, 父母与孩子  ☐ 祖父母, 父母, 孩子与帮佣  ☐ 其他, 请注明: ______________________________________________ |
| **最高学历** | ☐ 没有正规教育  ☐ 小学教育  ☐ 中学教育  ☐ 高等教育  ☐ 其他, 请注明: ______________________________________________  接受教育年数: ________________________________________________ |
| **房屋类型** | ☐ 公共房屋: 政府组屋  ☐ 私人房屋: 公寓, 套房, 有地住宅  ☐ 其他, 请注明: ______________________________________________ |
| **房屋空间** | ☐ 1至3房 ☐ 4至5房  ☐ 5房以上, 请注明: ___________________________________________ |
| **家庭每月平均工作的收入** | ☐ S$2,000以下 ☐ S$6,000 – $8,000  ☐ S$2,000 – $4,000 ☐ S$8,000 – $10,000  ☐ S$4,000 – $6,000 ☐ S$10,000以上 |
| **父母有糖尿病吗？** | ☐ 无 ☐ 双亲  ☐ 母亲 ☐ 父亲 |
| **父母有高血压吗？** | ☐ 无 ☐ 双亲  ☐ 母亲 ☐ 父亲 |
| **父母有高胆固醇吗？** | ☐ 无 ☐ 双亲  ☐ 母亲 ☐ 父亲 |
| **父母曾感染手足口症/疱疹性咽峡炎吗?** | ☐ 无 ☐ 双亲  ☐ 母亲 ☐ 父亲 |
| **孩子感染手足口症/疱疹性咽峡炎时平均休假的天数** | ☐ 1天 ☐ 5天以上  ☐ 2 – 4天 ☐ 0天 原因:___________________________ |
| **孩子感染手足口症/疱疹性咽峡炎时平均医疗咨询的花费** | ☐ S$50以下 ☐ S$100以上  ☐ S$51 – S$100 ☐ S$0 原因:___________________________ |

| **III. 风险因素** | |
| --- | --- |
| **孩子在户外游乐场玩耍** | ☐ 从没  ☐ 每2至3周一次  ☐ 一周一次  ☐ 每周不止一次  ☐ 每日 |
| **孩子在室内游乐场玩耍** | ☐ 从没  ☐ 每2至3周一次  ☐ 一周一次  ☐ 每周不止一次  ☐ 每日 |
| **孩子和邻近的其他孩子们一起玩耍** | ☐ 从没  ☐ 每2至3周一次  ☐ 一周一次  ☐ 每周不止一次  ☐ 每日 |
| **孩子在用餐前，用肥皂洗手** | ☐ 从没  ☐ 有时  ☐ 总是 |
| **孩子在用餐后，用肥皂洗手** | ☐ 从没  ☐ 有时  ☐ 总是 |
| **孩子上厕所后，用肥皂洗手** | ☐ 从没  ☐ 有时  ☐ 总是 |
| **大人替孩子换尿布或清洗上厕所的孩子后，用肥皂洗手** | ☐ 从没  ☐ 有时  ☐ 总是 |
| **大人在喂食孩子前，用肥皂洗手** | ☐ 从没  ☐ 有时  ☐ 总是 |
| **在外用餐时，让孩子使用消毒液消毒手** | ☐ 从没  ☐ 有时  ☐ 总是 |
| **在外用餐时，用消毒液消毒婴儿座椅** | ☐ 从没  ☐ 有时  ☐ 总是 |
| **在家里用肥皂清洗玩具** | ☐ 从没  ☐ 每2至3周一次  ☐ 一周一次  ☐ 每周不止一次  ☐ 每日 |
| **在家里用消毒液消毒玩具** | ☐ 从没  ☐ 每2至3周一次  ☐ 一周一次  ☐ 每周不止一次  ☐ 每日 |
| **清洁家居的频率** | ☐ 从没  ☐ 每2至3周一次  ☐ 一周一次  ☐ 每周不止一次  ☐ 每日 |
| **在家里与兄弟姐妹共用餐具** | ☐ 从没  ☐ 有时  ☐ 总是 |

| **IV. 手足口症的常识** | |
| --- | --- |
| **手足口症通过直接接触感染者的鼻腔分泌物（如唾液，痰液或粘液），水疱或皮疹液体和粪便，传染给健康的人。** | ☐ 否  ☐ 是  ☐ 不知道 |
| **发烧，口腔溃疡，手脚掌和/或臀部的皮疹或水疱是手足口症的一些常见症状和体征。** | ☐ 否  ☐ 是  ☐ 不知道 |
| **手足口症的潜伏期（从感染到出现症状）通常为3至5天，可从2天至2周。** | ☐ 否  ☐ 是  ☐ 不知道 |
| **成年人可感染手足口症。** | ☐ 否  ☐ 是  ☐ 不知道 |
| **5岁以下的孩童感染手足口症的风险较高。** | ☐ 否  ☐ 是  ☐ 不知道 |
| **当孩子感染手足口症时，将孩子留在家中并远离公共场所。** | ☐ 否  ☐ 是  ☐ 不知道 |
| **良好的个人卫生对预防手足口症非常重要。** | ☐ 否  ☐ 是  ☐ 不知道 |
| **除了舒缓症状外，没有针对手足口症具体的治疗方法。** | ☐ 否  ☐ 是  ☐ 不知道 |
| **目前没有手足口症的疫苗。** | ☐ 否  ☐ 是  ☐ 不知道 |
| **当孩子被诊断感染手足口症时，立即通知孩子所在的幼儿中心。** | ☐ 否  ☐ 是  ☐ 不知道 |
| **只有在幼儿病假结束后并确认孩子已经没有任何手足口症的症状后，孩子才能返回幼儿中心。** | ☐ 否  ☐ 是  ☐ 不知道 |
| **不与他人共用食物/饮料，餐具，牙刷或毛巾以预防手足口症的传染。** | ☐ 否  ☐ 是  ☐ 不知道 |
| **对被鼻腔或口腔分泌物污染的玩具或器具进行彻底消毒。** | ☐ 否  ☐ 是  ☐ 不知道 |
| **即使孩子已经康复，手足口症能持续通过孩子的粪便传染长达12周。** | ☐ 否  ☐ 是  ☐ 不知道 |
| **即使孩子已经康复，手足口症能持续通过孩子的唾液传染长达4周。** | ☐ 否  ☐ 是  ☐ 不知道 |

**------------------------------------------------------------------完------------------------------------------------------------------**
